# Global change and ecosystem connectivity: How geese link fields of central Europe to eutrophication of Arctic freshwaters

**DOI:** 10.1007/s13280-016-0802-9

**Published:** 2016-06-28

**Authors:** Dag O. Hessen, Ingunn M. Tombre, Gerben van Geest, Kristian Alfsnes

**Affiliations:** 1 Section for Aquatic Biology and Toxicology (AKVA), Department of Biosciences, University of Oslo, 0316 Oslo, Norway; 2 Department of Arctic Ecology, The Fram Centre, Norwegian Institute for Nature Research, P.O. Box, 9296 Tromsø, Norway; 3 Department of Ecology and Water Quality, Deltares, P.O. Box 177, 2600 MH Delft, Netherlands; 4 Faculty of Science Aquatic Ecology and Environmental Biology, Radboud University, P.O. Box 9010, 6500 GL Nijmegen, Netherlands; 5 Department of Aquatic Ecology, Netherlands Institute for Ecology (NIOO), Droevendaalsesteeg 10, 6708 PB Wageningen, Netherlands

**Keywords:** Arctic, Connectivity, Eutrophication, Migration

## Abstract

Migratory connectivity by birds may mutually affect different ecosystems over large distances. Populations of geese overwintering in southern areas while breeding in high-latitude ecosystems have increased strongly over the past decades. The increase is likely due to positive feedbacks caused by climate change at both wintering, stopover sites and breeding grounds, land-use practices at the overwintering grounds and protection from hunting. Here we show how increasing goose populations in temperate regions, and increased breeding success in the Arctic, entail a positive feedback with strong impacts on Arctic freshwater ecosystems in the form of eutrophication. This may again strongly affect community composition and productivity of the ponds, due to increased nutrient loadings or birds serving as vectors for new species.

## Introduction

Ecosystems are rarely closed entities, and with few exceptions like islands, lakes, isolated forests and mountain areas, boundaries are often arbitrarily defined. Moreover, even lakes and islands are clearly affected by their surroundings and neighbouring ecosystems. For rivers, the concept of ecosystem connectivity or donor-fed systems originates from the observation that catchment properties affect recipient systems in fundamental ways (Polis et al. 1997; Bartels 2012), which also holds for lakes (Cloern 2007; Soininen et al. 2015). Aquatic ecosystem connectivity often deals with adjacent ecosystems, e.g. where litterfall or dissolved organic matter from catchments may serve as an energy subsidy to aquatic systems (Jansson et al. 2007; Bartels 2012; Soininen et al. 2015).

Ecosystems may also be connected over long distances. Migratory animals often represent the most conspicuous and long-range type of ecosystem connectivity both with regard to nutrients, organic matter, toxicants, propagules, parasites and pathogens, as well as by direct or indirect trophic effects (Bauer and Hoye 2014). For aquatic ecosystems, migrating fish often represents major fluxes of energy and nutrients, e.g. post-spawning carcasses from anadromous salmon-fertilizing rivers or rivers banks (Cederholm et al. 1999). In such cases, there is also a feedback component involved, since litter fall (from land) may promote survival and growth of fish fry. This may also be linked to trophic cascades within the ecosystem, where fertilization may boost autotroph production, propagating up the trophic ladder (cf. Ripple et al. 2001). Also birds may constitute important links between distant ecosystems (Webster et al. 2001; Jefferies et al. 2004a, b), especially in the context of nutrient loads (van Geest et al. 2007; Hahn et al. 2008; Dessborn et al. 2016).

The major transitions or degradation of ecosystems worldwide, combined with climate change and change in population size of many migrating animals may affect ecosystems profoundly (Bauer and Hoye 2014; Doughty et al. 2016). Here we will use goose migration and Arctic freshwater ecosystem impact as an illustration of this interplay between changed climate and management regimes, and how it may affect properties of distant ecosystems.

## States of ecosystem connectivity

**Fig. 1:**
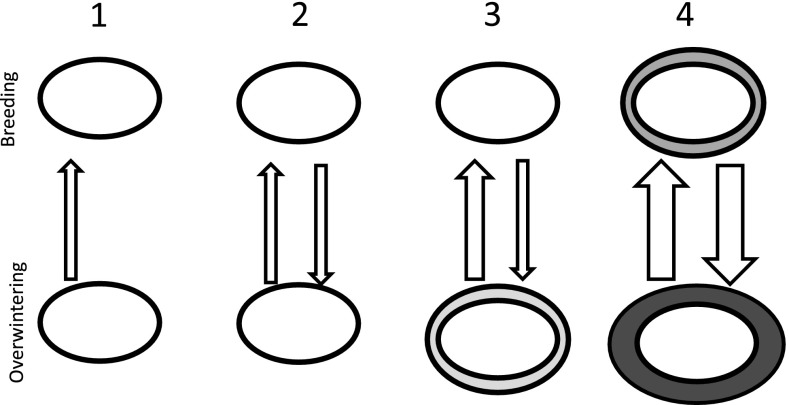
A conceptual model demonstrating connectivity and feedbacks between systems. *Circles* represent ecosystems, areas or populations, whereas the *thickness of the arrows* indicates different levels of influence. *Enlarged circles* represent systems affecting other systems by, e.g. increased population size and/or high degree of mutual influence. (1) One-sided effects: a simple donor and recipient scenario without feedbacks. (2) Mutual effects, control mechanisms operating at both sites: a steady-state system with mutual feedbacks between systems. (3) Mutual effects, control at one site: a feedback system where the original donor system (*lower panel*) is impacted by e.g. increased productivity or population size (e.g. by climate change or fertilization). (4) Mutual effects at both sites: a non-equilibrium feedback situation where increased productivity or population in both systems pose a mutual stimulation. In the case of the goose-Arctic lake system, the *lower panel* may represent overwintering grounds in central Europe, while the *upper panel* represents breeding grounds in the high Arctic

Ecosystem connectivity may have different regulating mechanisms and outcomes, conceptually illustrated as four possible cases in Fig. 1. In the first case (1), there is a predominant one-way flow of energy or nutrients from a donor system to a recipient, e.g. terrestrial flux of organic matter from terrestrial catchment to rivers, lakes or coast (Soininen et al. 2015). Another example is the seabird-mediated fertilization on land (Anderson and Polis 1999) or anadromous fish (Cederholm et al. 1999). In both cases, there are negligible feedbacks on the marine system. In the next case (2), both systems are significantly affected by each other (although not necessarily equally so). Seasonal migration between breeding, spawning or overwintering areas, would serve as typical examples of systems with mutual feedback. Under stable conditions, a kind of long-term equilibrium of population size of the species involved could be established. The processes controlling the population could either occur at the site of reproduction, at the overwintering area or during the migration, and while there clearly is inter-annual variability, there are no systematic changes in population size (e.g. bird migration in unmanaged systems). Even if one site is released from population control by increased productivity, decreased harvesting or management practices, the population size may be regulated by the other “bottleneck” site, e.g. anadromous fish with restricted spawning grounds. Also in the case of decimation or habitat deterioration, exemplified in (3), this may be counteracted by e.g. improved breeding success of the remaining individuals thus serving as a donor site for maintenance of population size. In cases, however, where both systems are released from control or positively stimulated, like in (4), a kind of positive feedback loop may be operating with potentially strong, and unexpected, ecosystem impacts, eventually approaching a new equilibrium state. The positive feedback in this context is strictly on the population size where both systems act as reciprocally donor system. For example, in the case of arctic-nesting geese, an initially increased breeding success implies a larger population, and with improved conditions also in the wintering site, a larger fraction of the population will survive and migrate back to the breeding site. The release of regulation mechanisms is likely to differ between both sites, and could be caused by a reduced predation, harvesting or hunting, increased productivity and food access or improved habitat or habitat range mediated by for example climate change.

It should be stressed that there are gradual transitions from (1) to (4), and this is not an exhaustive list of types of connectivity. There has been a dramatic decline of many animal populations worldwide, which may have a huge impact on global rates of nutrient transport (Doughty et al. 2016). Additionally, structural changes in ecosystems with loss of apex predators may have cascading effects down the trophic ladder (Strong and Frank 2010) and may also affect migrating species both positively and negatively. While climate change is likely to impose further constraints on many species and populations, it may however also in cases promote population increase and give some literally far-reaching and unforeseen consequences. Below we will describe the development of the geese breeding in the high-arctic archipelago of Svalbard, a typical example of this scenario, and also point to the severe ecosystem impacts in the high Arctic.

## The Svalbard case

**Fig. 2:**
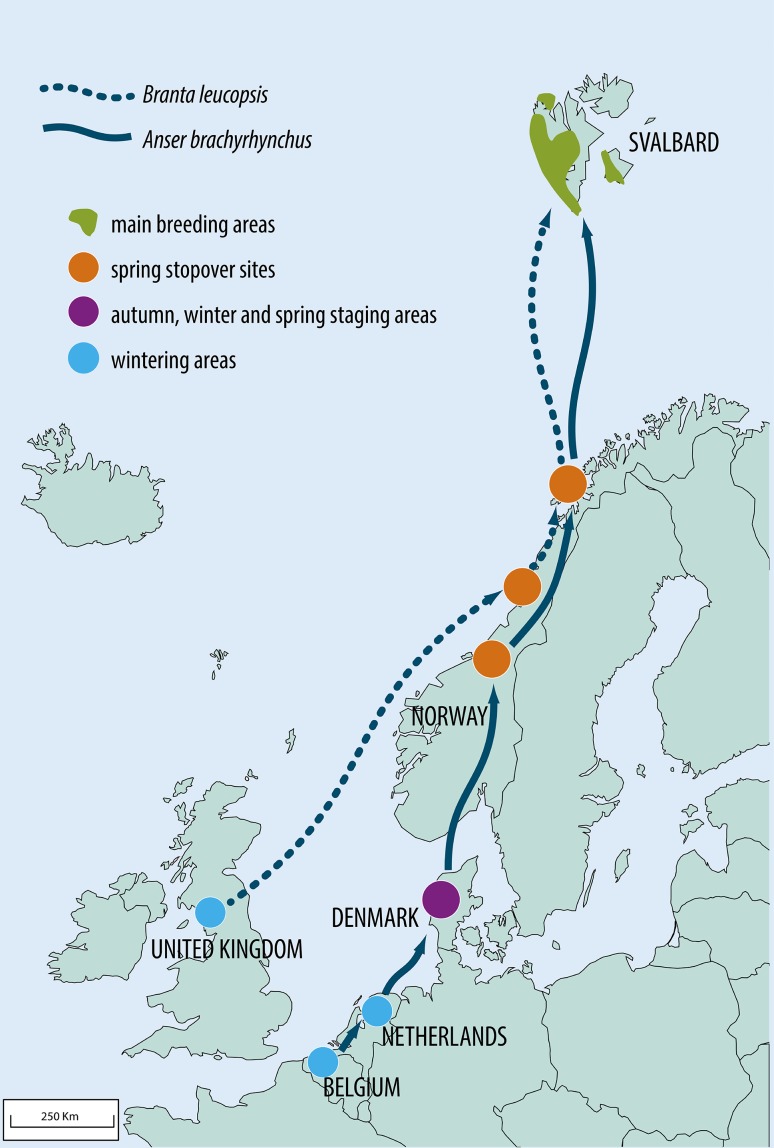
Map of the flyways for two breeding populations of geese in Svalbard, the pink-footed goose *Anser brachyrhynchus* and the barnacle goose *Branta leucopsis*. Wintering sites, spring stopover sites and breeding ground are shown

Migratory connectivity mediated by birds is common in northern latitudes (Schmiegelow and Mönkkönen 2002; Webster et al. 2002). Especially, the increasing populations of large grazers like geese (Madsen et al. 1999; Fox 2010; Pedersen et al. 2013a, b) may have profound ecosystem impacts at their breeding areas far away from their wintering areas (Jefferies et al. 2004a, b, 2006, Jefferies 2006; Van der Wal et al. 2007). Mobile consumers, such as birds, may provide substantial contributions to local nutrient cycles (Hahn et al. 2007, 2008). Because waterfowl aggregate in large groups in wetlands, nutrient load derived from guano may contribute up to 30–60 % of nutrient loading rates in certain wetland areas (Post 2008). These bird-borne nutrients may cause eutrophication of wetlands (Dessborn et al. 2016), potentially resulting in changes in physicochemical properties and community composition. At high latitudes with low terrestrial productivity, seabirds are often instrumental for providing nutrient inputs to terrestrial productivity (Odasz 1994; Anderson and Polis 1999; Hop et al. 2006). In this context, we will link a well-documented story of increasing populations of arctic-breeding geese, to its less recognized, but remarkable impact on Arctic lakes and ponds in the high-Arctic archipelago of Svalbard. Two of the goose species breeding in Svalbard, the pink-footed goose (*Anser brachyrhynchus* Baillon) and the barnacle goose (*Branta leucopsis* Bechstein) spend their winter in temperate regions in Europe and have increased over the past decades (Fig. 2). As they connect the temperate and arctic regions via their yearly migration to the breeding grounds, regulating mechanisms and outcome in different types of connectivity patterns can be evaluated.

## Drivers and development of Arctic goose populations

**Fig. 3:**
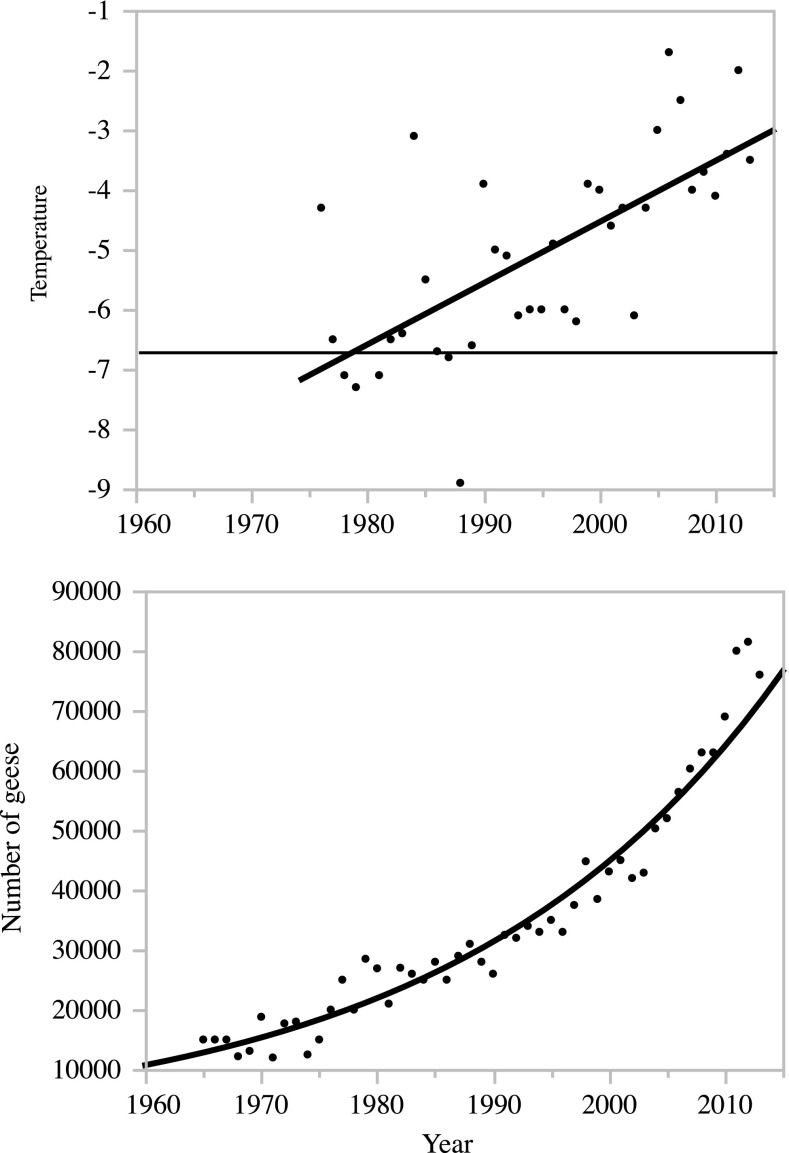
Elevated temperature and the development of the pink-footed goose population from 1960 to 2013. Temperature over years represented as linear regression (*p* < 0.0001, *r* ^2^ = 0.46, *F* ratio 29.64); Goose numbers over years given by 2. degree, quadratic polynomial curve fit (*p* < 0.0001, *r* ^2^ = 0.95, *F*-ratio = 445.77).Temperature data obtained from the Norwegian Meteorological Institute, goose data from Madsen et al. (2013)

The Svalbard-breeding populations of barnacle geese and pink-footed geese have increased dramatically during the last decades (Fox 2010; Madsen et al. 2013). This accompanies a striking increase in annual average temperature at the archipelago, and long-term monitoring at two western stations in the regions where there are high densities of breeding geese, reveals an annual increase in temperature of approximately 3 °C over the past 40 years (Fig. 3). The earlier snowmelt, and extended breeding season and breeding range promoted by this climatic trend in the high Arctic, as well as extended growing seasons and spring temperature along the spring stopover sites will have a positive impact on the goose populations (Prop et al. 1998; Van Eerden 2005; Madsen et al. 2007; Jensen et al. 2014). As a result, the population of pink-footed geese have increased 7-fold over the same period (Madsen and Williams 2012; Madsen et al. 2013), whereas the barnacle goose population has increased more than three times (Fox 2010; Griffin 2014). The populations’ increase may be accredited to a combination of protection from hunting, increased winter survival due to improved food availability and quality caused by the shifts and intensified agricultural practice, and, finally, a warmer climate along stopover sites and at the breeding grounds (Van Roomen and Madsen 1991; Ebbinge 1992; Madsen et al. 1999; Fox et al. 2005).

In temperate regions, agricultural schemes have been used as a “green policy” (Madsen et al. 2014) providing agricultural land to grazing geese (Owen 1977; van Eerden 1990; Patterson and Fuchs 2001; Tombre et al. 2013), further increasing the survival of the European goose populations. For the Svalbard barnacle geese, most of the wintering areas in UK are protected agricultural land (Cope et al. 2003), being one of the main reasons for the population’s success (Owen 1977). For the pink-footed geese, the improved climate on the nesting grounds at Svalbard has increased their breeding success significantly over the last decade (Madsen et al. 2007; Jensen et al. 2014). More pink-footed goose pairs are able to find nest sites within the narrow time window, characteristic for the arctic-breeding conditions. A series of seasons with early snowmelt has caused an almost exponential increase for this population over the past decade (Fig. 3, Madsen and Williams 2012; Madsen et al. 2013). Earlier spring development, and thus an extended growing season, is likely to continue over the coming decades as judged from climate scenarios (Førland et al. 2011). Accordingly, this may also expand the distribution of the goose species in Svalbard, as has been predicted for the pink-footed goose population (Jensen 2008; Wisz et al. 2008). Future scenarios for goose population sizes however not only depend on direct effect of warmer climate and extended growing seasons, but also on indirect effects, e.g. polar bears have increased the goose egg-predation rate in Svalbard (Prop et al. 2015), farmland practices in overwintering areas and stopover sites as well as management actions in the form of increased hunting pressure may also reduce the survival rate for geese on the long term (Madsen and Williams 2012).

Regardless of future population scenarios, a large number of geese are at present affecting surface waters at their breeding grounds in Svalbard. They release nutrients in the watersheds and directly in the water bodies, and in Svalbard such ponds are mostly shallow permafrost ponds in coastal areas where the geese breed and graze (van Geest et al. 2007). The goose-mediated effect will add to the direct stimulatory effects of climate change for primary production in arctic lakes and ponds, due to warming of the ponds (Quinlan et al. 2005; Smol and Douglas 2007) as well as climate impacts to the surrounding soil and plant communities, resulting in increased fluxes of terrestrial organic matter and nutrients to the ponds (Luoto et al. 2015; Smol et al. 2005). Geese may also strongly affect community composition and food web structure of the Arctic freshwater by potentially serving as vectors for spreading of invertebrates, plants and microorganisms (cf. Green 2002; Figureola and Green 2002). This implies linkages between freshwater and terrestrial environments, and demonstrates that ecosystem effects at lower latitudes, i.e. increased survival of geese during winter and migration, may have local consequences in arctic ecosystems.

For the case with geese and ponds in Svalbard, the patterns in connectivity between temperate and arctic regions have shifted from cases (2) to (4) over the last decades (cf. Fig. 1). Goose numbers were previously controlled both by restricted breeding areas, short breeding seasons, winter mortality, but as the conditions have improved, survival and breeding rates have increased, so will the impacts on arctic ponds in the form of fertilization from larger goose populations also increase.

## Goose-promoted eutrophication in the arctic

**Fig. 4:**
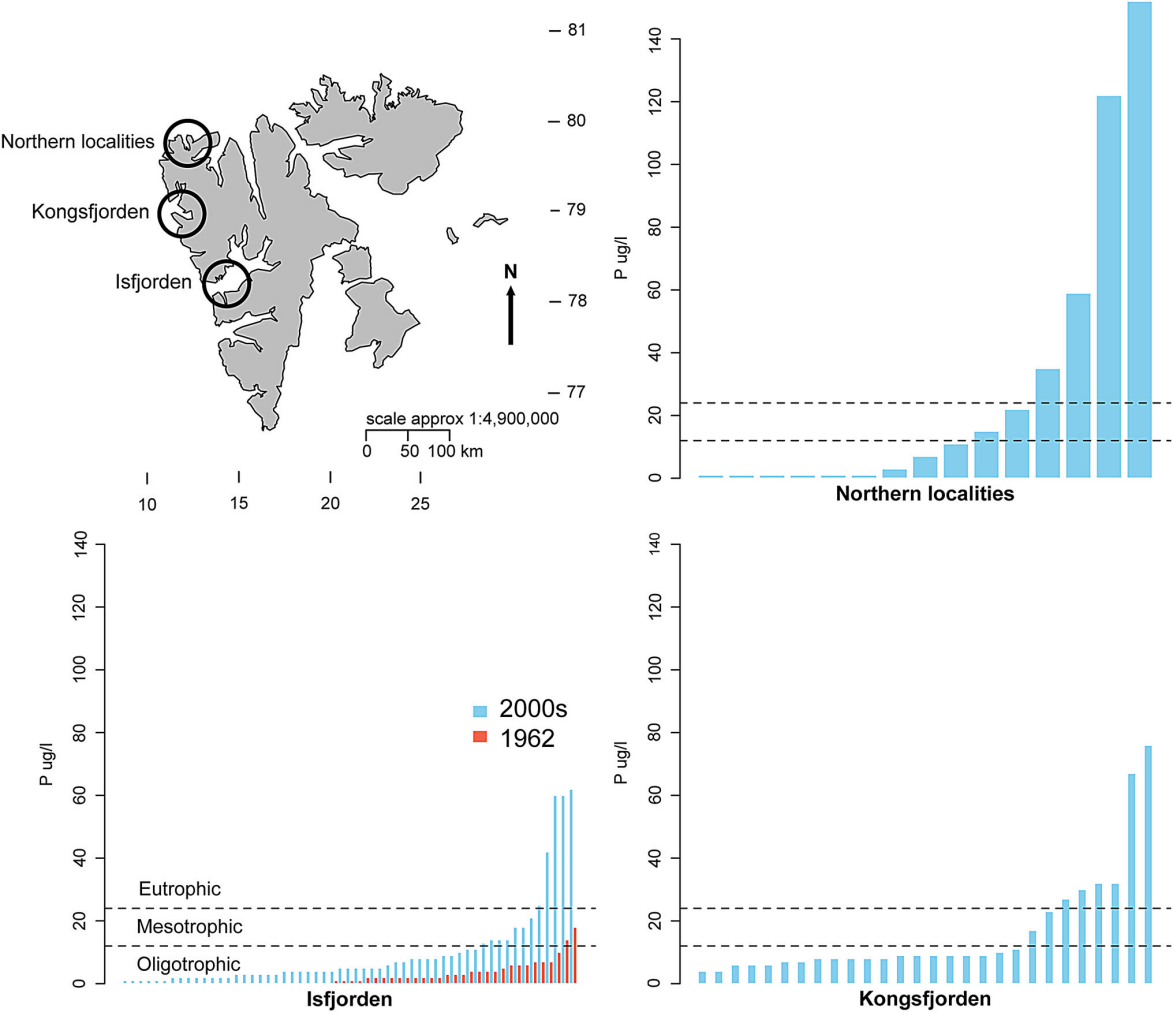
Map of Svalbard with sampling areas and major goose area hatches, and *bar charts* for P levels in the three sampled regions (name of locations in *parenthesis*): Isfjorden (Cape Linné, Nordenskioldkysten, Erdmannsvatna), Kongsfjorden (Ny-Ålesund), and Northern localities (Danskøya, Reinsdyrflya, Måkeøyane)

Most of the Svalbard localities are naturally poor in nutrients, but there have clearly been sites where impacts from guano have been prevalent historically. The assessment of water quality impact by the increasing goose populations is somewhat hampered by the lack of corresponding time series, but there are a few systematic samples of lakes and ponds at these latitudes that offer reliable “background” nutrient analysis. A comprehensive survey of plankton was yearly conducted in 1959–1962 in the Isfjorden area (Amrén 1964a, b; Willén 1980), and a large number of freshwater localities have been sampled for nutrient analysis and zooplankton species composition in different regions of Svalbard also in recent years; July–August in 2003, 2004, 2008 and 2014 (Fig. 4). For the 2008-data, the 48 ponds sampled almost completely overlap with the survey of 1959–1962. Locations for sampling were primarily chosen because they possess a range of freshwater localities, and they are sites either known as traditional sites used by geese and/or being locations with expanding goose populations (Wisz et al. 2008; Tombre et al. 2012). Most localities have depths <2 m, freeze solid during winter, and are thus devoid of fish.

The surveyed ponds in recent years were distributed over three main areas: Isfjorden, Kongsfjorden and Northern localities (Fig. 4). They displayed a wide span in nutrient concentrations, ranging from <1 (detection limit) up to 60 µg Phosphorus l^−1^ at the Isfjorden sites, from 5 to almost 80 μg P l^−1^ in the Ny-Ålesund area in Kongsfjorden, while the northernmost sites spanned from <1 to almost 150 µg P l^−1^. During surveys in 2003–2014, geese exerted a variable impact on the ponds at all these sites, and comparisons between bird impacted and non-impacted ponds gave strong evidence of a eutrophication mediated by birds (van Geest et al. 2007: Alfsnes et al. 2016).

From this previous survey, average P was 4.3 µg P l^−1^, and the maximum was 18 µg P l^−1^. Hence, the average concentrations of P from this area have increased fourfold over 50 years compared to the samples in 1962, and a close association between high levels of nutrients and visual signs of goose activity (droppings, feathers) has been reported from the area (van Geest et al. 2007).

Also for the Kongsfjorden area and the Northern localities, the most eutrophied sites had the most prominent signs of geese (or other birds) in terms of droppings and feathers at the shores (van Geest et al. 2007; Alfsnes et al. 2016). Hence, it is likely that birds, and in most cases geese, were the primary source of nutrients to these localities. Moreover, the increasing numbers of geese have also influenced properties of the water bodies by providing organic carbon via droppings, which changes the vegetation cover, which again changes the runoff. In a study by van Geest et al. (2007), it was also demonstrated that molar N:P-ratio of fresh droppings on the ground from barnacle geese was in the range of 6–9. In ponds where N was analysed, it appeared to be closely correlated with P, indicating that P mostly had a biotic origin (i.e. not related to inorganic clay particles).

Increased nutrient concentrations may not necessarily result in a higher standing stock of phytoplankton, since a large fraction of primary producers in these systems are benthic algae (Rautio and Vincent 2006). Moreover, the fact that zooplankton grazers in all these fishless systems constitute the top trophic levels implies a strong grazing pressure and low autotroph biomass in the open waters, since nutrients are channelled into zooplankton (Van Geest et al. 2007; Van der Wal and Hessen 2009). Hence, the fertilization may indirectly affect not only productivity, but also shifts in the relative abundance of species in the community by promoting more nutrient-demanding species of both autotrophs and heterotrophs.

Another consequence of increased bird migration is the potential of transporting zooplankton resting eggs, or stages, via gut content or feathers on geese. It may promote the establishment of invertebrate invaders and infectious diseases (bacteria, fungi, unicellular parasites) both between Svalbard localities and potentially also from mainland Europe to the Arctic. While we still have insufficient data to actually link species shifts and new species to birds or climate changes (or a combination of both), this will be an important task to address in the future. There are, however, already at present pronounced differences in both clonal and species composition of *Daphnia* in ponds with different nutrient status, which may be related to the impact of geese (Van Geest et al. 2007; Alfsnes et al. 2016). If larger parts of the high Arctic become a pre-breeding area for geese (Hubner 2006), and there will be an expansion of the breeding distribution (Wisz et al. 2008), this will increase the probability both for bird-induced dispersal of zooplankton species and community shifts due to eutrophication.

Collectively, our synthesis demonstrates how changes in climate and land use in terrestrial ecosystems in Central Europe may have far-reaching consequences for “pristine” and completely different ecosystems thousands of kilometres further north. The improved conditions in the high Arctic (from a goose perspective), partly related to climate change and extended growth season, serve as a feedback affecting the overwintering habitats in terms of more geese. The development and impacts reported in the present study have some similarities to those reported on the North American continent, where increasing numbers of snow geese (*Anser caerulescens caerulescens*) have resulted from improved overwintering conditions. This cause a set of impacts on the salt marches at the arctic La Pérouse Bay (Canada) due to intensified grazing, grubbing and nutrient cycling (Jefferies et al. 2004a, b), demonstrating a shift from case (2/3) to case (4) as described in Fig. 1. At the Svalbard sites, there are signs of grubbing and grazing by geese on the tundra (Van der Wal et al. 2007; Speed 2009), but even more striking are the impacts on the freshwater ecosystems in the form of nutrient enrichment. This demonstrates the often unforeseen and complex effects of climate and land use on ecosystems due to ecosystem connectivity, highlighting the need for integrated and international ecosystem management.

## References

[CR2] Alfsnes, K., A. Hobæk, L.J. Wieder, and D.O. Hessen. 2016. Birds, nutrients and climate change: mtDNA haplotype diversity of Arctic *Daphnia* on Svalbard revisited. Polar Biology. doi:10.1007/s00300-015-1868-8.

[CR3] Amrén H (1964). Ecological studies of zooplankton populations in some ponds of Spitsbergen. Zoologiska bidrag från Uppsala.

[CR4] Amrén H (1964). Ecological and taxonomical studies on zooplankton from Spitsbergen. Zoologiska bidrag från Uppsala.

[CR5] Anderson WB, Polis GA (1999). Nutrient fluxes from water to land: Seabirds affect plant nutrient status on Gulf of California islands. Oecologia.

[CR6] Bartels P (2012). Reciprocal subsidies between freshwater and terrestrial ecosystems structure consumer resource dynamics. Ecology.

[CR7] Bauer S, Hoye BJ (2014). Migratory animals couple biodiversity and ecosystem functioning worldwide. Science.

[CR9] Cederholm MD, Kunze MD, Murota T, Sibatani A (1999). Pacific salmon carcasses: Essential contributions of nutrients and energy for aquatic and terrestrial ecosystems, Terrestrial carbon and intraspecific size-variation shape lake ecosystems. Fisheries.

[CR10] Cloern JP (2007). Habitat connectivity and ecosystem productivity: Implications from a simple model. American Naturalist.

[CR11] Cope DR, Pettifor RA, Griffin LR, Rowcliffe JM (2003). Integrating farming and wildlife conservation: The Barnacle Goose Management Scheme. Biological Conservation.

[CR13] Dessborn RH, Hessel R, Elmberg J (2016). Geese as vectors of nitrogen and phosphorus to freshwater systems. Inland Waters.

[CR14] Doughty CE, Roman J, Faurby S, Wolf A, Haque A, Bakker ES, Malhi Y, Dunning JB (2016). Global nutrient transport in a world of giants. Proceedings of the National Academy of Sciences.

[CR15] Ebbinge BS (1992). Population limitation in arctic-breeding geese.

[CR16] Figureola J, Green AJ (2002). Dispersal of aquatic organisms by waterbirds: A review of past research and priorities for future studies. Freshwater Biology.

[CR17] Førland, E.J., R. Benestad, I. Hansen-Bauer, J.E. Haugen, and T.E. Skaugen. 2011. Temperature and precipitation development at Svalbard 1900–2100. Adv. Meteorol. doi:10.1155/2011/893790.

[CR18] Fox AD (2010). Current estimates of goose population sizes in Western Europe, a gap analysis and an assessment of trends. Ornis Svecica.

[CR19] Fox AD, Madsen J, Boyd H, Kuijken K, Norriss DW, Tombre IM, Stroud DA (2005). Effects of agricultural change on abundance, fitness components and distribution of two arctic-nesting goose populations. Global Change Biology.

[CR20] Green AJ (2002). Implications of waterbird ecology for the dispersal of aquatic organisms. Acta Oecologia.

[CR21] Griffin L (2014). Svalbard Barnacle Goose distribution around the Solway Firth 2013-2014: Flock counts from the Solway goose Management Scheme area.

[CR22] Hahn S, Bauer S, Klaassen M (2008). Quantification of allochthonous nutrient inputs into freshwater bodies by herbivorous waterbirds. - Freshw. Biol..

[CR23] Hop H, Pearson T, Hegseth EN, Kovacs KM, Wiencke C, Kwasniewski S, Eiane K, Mehlum F (2006). The marine ecosystem of Kongsfjorden, Svalbard. Polar Research.

[CR24] Hubner CE (2006). The importance of pre-breeding areas for the arctic barnacle goose *Branta leucopsis*. Ardea.

[CR25] Jansson M, Persson L, De Roos AM, Jones RI, Tranvik LJ (2007). Terrestrial carbon and intraspecific size-variation shape lake ecosystems. TREE.

[CR26] Jefferies RL (2006). A biotic agent promotes large-scale catastrophic change in the coastal marshes of Hudson Bay. Journal of Ecology.

[CR27] Jefferies RL, Rockwell RF, Abraham KF (2004). Agricultural food subsidies, migratory connectivity and large-scale disturbance in arctic coastal systems: A case study. Integrative and Comparative Biology.

[CR28] Jefferies RL, Rockwell RF, Abraham KF (2004). The embarrassment of riches: Agricultural food subsidies, high goose numbers, and loss of Arctic wetlands—a continuing saga. Environmental Reviews.

[CR29] Jefferies RL, Drent RH, Bakker JP (2006). Connecting Arctic and temperate wetlands and agricultural landscapes: The dynamics of goose populations in response to global change. Ecologcial Studies.

[CR30] Jensen RA (2008). Prediction of the nesting distribution of pink-footed geese (*Anser brachyrhynchus*) in Svalbard under a warmer climate scenario. Global Change Biology.

[CR31] Jensen GH, Madsen J, Johnson FA, Tamstorf MP (2014). Snow conditions as an estimator of the breeding output in high-Arctic pink-footed geese *Anser brachyrhynchus*. Polar Biology.

[CR33] Luoto TP, Oksman M, Ojala AEK (2015). Climate change and bird impact as drivers of high Arctic pond deterioration. Polar Biology.

[CR34] Madsen, J., G. Cracknell, and A.D. Fox (eds). 1999. *Goose populations of the western Palearctic. A review of status and distribution*, 344 pp. Wetlands International Publication No. 48. Wageningen/Rønde: Wetlands International/National Environmental Research Institute.

[CR35] Madsen, J., and J.J. Williams (eds). 2012. International Species Management Plan for the Svalbard Population of the Pink-footed Goose *Anser brachyrhynchus*. AEWA Technical Series No. 48. Bonn, Germany.

[CR36] Madsen, J., F. Cottaar, P.I. Nicolaisen, I. Tombre, C. Verscheure, and E. Kuijken. 2013. Svalbard Pink-footed Goose. Population Status Report 2012–2013. Technical Report from DCE—Danish Centre for Environment and Energy, No. 29, Aarhus University, 8 pp. ISBN 978-87-7156-039-8. http://pinkfootedgoose.aewa.info/publications.

[CR37] Madsen J, Tamstorf M, Klaassen M, Eide N, Glahder C, Rigét F, Nyegaard H, Cottaar F (2007). Effects of snow cover on the timing and success of reproduction in high-Arctic pink-footed geese *Anser brachyrhynchus*. Polar Biology.

[CR38] Madsen J, Bjerrum M, Tombre IM (2014). Regional management of farmland feeding geese using an ecological prioritization tool. Ambio.

[CR39] Odasz AM (1994). Nitrate reductase activity in vegetation below an arctic bird cliff, Svalbard, Norway. Journal of Vegetation Science.

[CR40] Owen M (1977). The role of wildfowl refuges on agricultural land in lessening the conflict between farmers and geese in Britain. Biological Conservation.

[CR41] Patterson IJ, Fuchs RME (2001). The use of nitrogen fertilizer on alternative grassland feeding refuges for pink-footed geese in spring. Journal of Applied Ecology.

[CR42] Pedersen ÅØ, Speed JDM, Tombre IM (2013). Prevalence of pink-footed goose grubbing in the arctic tundra increases with population expansion. Polar Biology.

[CR43] Pedersen ÅØ, Tombre I, Jepsen JU, Eidesen PB, Fuglei E, Stien A (2013). Spatial patterns of goose grubbing suggest elevated grubbing in dry habitats linked to early snow melt. Polar Research.

[CR44] Polis GA, Anderson WB, Holt RD (1997). Towards an integration of landscape and food web ecology: The dynamics of spatially subsidized food webs. Annual Review of Ecology, Evolution, and Systematics.

[CR45] Post DM (2008). The role of migratory waterfowl as nutrient vectors in a managed wetland. Conservation Biology.

[CR46] Prop J, Black JM, Shimmings P, Owen M (1998). The spring range of barnacle geese *Branta leucopsis* in relation to changes in land management and climate. Biological Conservation.

[CR47] Prop J, Aars J, Bårdsen B-J, Hanssen SA, Bech C, Bourgeon S, de Fouw J, Gabrielsen GW (2015). Climate change and the increasing role of polar bears on bird populations. Frontiers in Ecology and the Environment.

[CR48] Quinlan R, Douglas MSV, Smol JP (2005). Food web changes in arctic ecosystems related to climate warming. Global Change Biology.

[CR49] Rautio M, Vincent WF (2006). Benthic and pelagic food resources for zooplankton in shallow high-latitude lakes and ponds. Freshwater Biology.

[CR50] Ripple WJ, Larsen EJ, Renkin RA, Smith DW (2001). Trophic cascades among wolves, elk and aspen on Yellowstone National Park’s northern range. Biological Conservation.

[CR51] Schmiegelow FKA, Mönkkönen M (2002). Habitat loss and fragmentation in dynamic landscapes: Avian perspectives from the boreal forest. Ecological Applications.

[CR52] Smol JP, Douglas MSV (2007). Crossing the final ecological threshold in high Arctic ponds. Proceedings of the National Academy of Sciences.

[CR53] Smol JP, Wolfe AP, Birks HJB, Douglas MSV, Jones VJ, Korhola A, Pienitz R, Rühland K (2005). Climate-driven regime shifts in the biological communities of Arctic lakes. Proceedings of the National Academy of Sciences.

[CR54] Soininen J, Bartels JP, Heino J, Luoto M, Hillebrand H (2015). Towards more integrated ecosystem research in aquatic and terrestrial environments. BioScience.

[CR55] Speed JDM (2009). Predicting habitat utilization and extent of ecosystem disturbance by an increasing herbivore population. Ecosystems.

[CR56] Strong DR, Frank KT (2010). Human Involvement in food webs. Annual Review of Environment and Resources.

[CR57] Tombre, I.M., J. Madsen, P. Clausen, J. Prop, and F. Hanssen. 2012. GOOSEMAP: Site-specific information for geese occurring on Svalbard. http://goosemap.nina.no/goosemap_eng/Startpage.aspx.

[CR58] Tombre IM, Eythórsson E, Madsen J (2013). Towards a solution to the goose-agriculture conflict in north Norway, 1988–2012: The interplay between policy, stakeholder influences and goose population dynamics. PLOS ONE.

[CR59] Van der Wal R, Hessen DO (2009). Analogous aquatic and terrestrial food webs in the high Arctic: The structuring force of a harsh climate. Perspectives in Plant Ecology, Evolution and Systematics.

[CR60] Van der Wal R, Sjögersten S, Woodin SJ, Cooper EJ, Jónsdóttir IS, Kuijper D, Fox TAD, Huiskes AD (2007). Spring feeding by pink-footed geese reduces carbon stocks and sink strength in tundra ecosystems. Global Change Biology.

[CR61] Van Eerden MR (1990). The solution of goose damage problems in the Netherlands, with special reference to compensation schemes. Ibis.

[CR62] Van Eerden MR (2005). Connecting seas: Western Palearctic continental flyway for water birds in the perspective of changing land use and climate. Global Change Biology.

[CR63] Van Geest GJ, Hessen DO, Spierenburg P, Dahl-Hansen GAP, Christensen G, Faeroving PJ, Brehm M, Loonen MJJE (2007). Goose-mediated nutrient enrichment and planktonic grazer control in Arctic freshwater ponds. Oecologia.

[CR64] Van Roomen, M., and J. Madsen (Eds.). 1991. *Waterfowl and agriculture: Review and future perspectives of the crop damage conflict in Europe*. IWRB Special Publication No. 21. Slimbridge: International Waterfowl and Wetlands Research Bureau.

[CR65] Wisz MS, Tamstorf MP, Madsen J, Jespersen M (2008). Where might the western Svalbard tundra be vulnerable to pink-footed goose (*Anser brachyrhynchus*) population expansion? Clues from species distribution models. Diversity and Distributions.

